# Biosynthesis and Metabolic Engineering of Anthocyanins in *Arabidopsis thaliana*

**DOI:** 10.2174/1872208307666131218123538

**Published:** 2014-01

**Authors:** Ming-Zhu Shi, De-Yu Xie

**Affiliations:** Department of Plant and Microbial Biology, North Carolina State University, Raleigh, NC 27695, USA

**Keywords:** Anthocyanins, Arabidopsis thaliana, biosynthetic pathway, structural diversity, transcriptional regulation.

## Abstract

*Arabidopsis thaliana* is the first model plant, the genome of which has been sequenced. In general, intensive studies on this model plant over the past nearly 30 years have led to many new revolutionary understandings in every single aspect of plant biology. Here, we review the current understanding of anthocyanin biosynthesis in this model plant. Although the investigation of anthocyanin structures in this model plant was not performed until 2002, numerous studies over the past three decades have been conducted to understand the biosynthesis of anthocyanins. To date, it appears that all pathway genes of anthocyanins have been molecularly, genetically and biochemically characterized in this plant. These fundamental accomplishments have made Arabidopsis an ideal model to understand the regulatory mechanisms of anthocyanin pathway. Several studies have revealed that the biosynthesis of anthocyanins is controlled by WD40-bHLH-MYB (WBM) transcription factor complexes under lighting conditions. However, how different regulatory complexes coordinately and specifically regulate the pathway genes of anthocyanins remains unclear. In this review, we discuss current progresses and findings including structural diversity, regulatory properties and metabolic engineering of anthocyanins in *Arabidopsis thaliana*.

## INTRODUCTION

Anthocyanins are a group of colorful and bioactive natural pigments with numerous important physiological and ecological functions in plants. In general, anthocyanins attract pollinators and seed dispersers, protect plants from high light irradiation and scavenge free radicals produced in cells under stress conditions [1-6]. In addition, anthocyanins have many promising benefits for human health. Numerous studies have demonstrated that anthocyanins have antioxidative, anti-inflammatory, anti-carcinogenic and anti-microbial activities, and can prevent against cardiovascular diseases and diabetes and improve vision [7-12]. A recent study showed that feeding mice with a diet supplemented with transgenic tomatoes rich in anthocyanins resulted in an extension of life span [13].

*Arabidopsis thaliana* is the first model plant, the genome of which has been sequenced. Over the past nearly three decades, intensive studies on this model plant have greatly updated our understandings in plant biology including the biosynthesis and functions of anthocyanins and other metabolites. In this report, we review and discuss the structural diversity, biosynthesis and metabolic engineering of anthocyanins in this model plant. 

## STRUCTURAL FEATURES OF ANTHOCYANINS

The study of anthocyanin biosynthesis has become one of the main focuses of the study of flavonoids in Arabidopsissince late 1980s. However, the structural properties of anthocyanins in Arabidopsis were unknown until 2002 when two anthocyanins were identified from leaf tissues [14]. Since then, new anthocyanin molecules have been continuously characterized, most of which were identified from *pap1-D* (*production of anthocyanin pigment 1-Dominant*) and *35S*:*PAP1* transgenic plants as well as red *pap1-D* callus cultures [15-18]. *PAP1* encodes a transcription factor that has been demonstrated to be a master regulator activating anthocyanin biosynthesis in Arabidopsis. The *pap1-D* and *35S*:*PAP1 *plants are featured by an enhanced accumulation of anthocyanins resulting from the overexpression of *PAP1* [19]. To date, more than twenty-nine anthocyanin molecules including *trans-* and *cis-* isomers have been identified from Arabidopsis (Table **1**; Fig. (**1**)), which are grown in different growth conditions such as high light intensities [16] and low temperature combined with high light [15].

Cyanidin has been identified as the predominant anthocyanidin aglycone in Arabidopsis. To date, all identified Arabidopsis anthocyanin molecules are derived from cyanidin through different modifications such as glycosylation, acylation and methylation, Fig. (**1**). These anthocyanin molecules are numerated as A1, A2, A3 and so on, in which “A” means “Anthocyanin” (Fig. (**1**); Table **1**). Anthocyanin profiles seem to differ in distinct tissues. For example, A11 appears to be the most abundant anthocyanin molecule in leaf tissues [14-16,18], while A5 is the most abundant one detected from roots [18]. Interestingly, anthocyanin molecules with a sinapoyl moiety (A4, A7, A9, A10 and A11) were not detected in roots [18] as well as in *pap1-D* callus cultures [17]. Several methylated anthocyanins (A14, A15, A16, A17, and A19) have been identified, but for most of them the methylation site in the structure has not been determined yet [16,17]. 

In addition, seedlings treated with anthocyanin precursors have been reported to form new anthocyanin molecules. Seedlings of both *Col* and *Ler* ecotypes treated with naringenin were able to synthesize cyanidin 3-*O*-glucosides (C3Gs) (449 m/z) and three unknown anthocyanin molecules featured by a mass spectrum of 611 m/z [23]. These four anthocyanin molecules are not detectable in plants in untreated conditions. This study indicates that the number and types of anthocyanins that can be produced by this model plant are likely more complicated than our current understanding. As more experiments are being continued, resulting data will enhance our understanding of the structural diversity of anthocyanin molecules in Arabidopsis.

## AGLYCONE STRUCTURE MODIFICATIONS

To date, all identified anthocyanin molecules of Arabidopsis are derived from side group modifications of cyanidin through mechanisms of glycosylation, acylation and/or methylation. These modifications have been reported to increase the stability of anthocyanins in aqueous solution and may likely alter their light absorption properties [24,25]. Eight genes have been isolated and biochemically characterized to be associated with these different modifications as described below (Table **2**). 

Glycosylation is one of the main biochemical mechanisms leading to diverse anthocyanin molecules in Arabidopsis. All anthocyanins identified in Arabidopsis contain at least one sugar group. The hydroxyl groups at C3 and C5 positions of cyanidin have been reported to be the two commonest targets of glucosylation [18,23,25]. These two glucosylation reactions have been characterized to be catalyzed by two major glucosyltransferases, UGT78D2 and UGT75C1, which are encoded by *At5g17050* and *At4g14090*, respectively [18]. UGT78D2 has been reported to glucosylate the hydroxyl group at C3 to form cyanidin 3-*O*-glucosides. In addition, this enzyme has been reported to catalyze the glycosylation of the hydroxyl group at C3 of flavonols and thus is called a flavonoid 3-*O*-glycosyltransferase. UGT75C1 has been reported to glucosylate the hydroxyl group at C5 to form cyanidin 5-*O*-glucosides. In cyanidin 3,5-*O*-glucosides, the glucosylation of the hydroxyl group at C3 has been reported to occur prior to that at C5 [18]. The formation of cyanidin 3-*O*-glucoside, cyanidin 5-*O*-glucoside and cyanidin 3, 5-*O*-glucoside most likely are the beginning steps of glycosylation. Subsequent glycosylations lead to more diverse and complex cyanin molecules in this plant. Two other glycosyltransferases encoded by *UGT79B1 *and *UGT84A2* respectively were recently identified to be involved in subsequent glycosylation of cyanidin 3-*O*-glucosides [26]. UGT79B1 is a cyanidin 3-*O*-glucoside: 2’’-*O*-xylosyltransferase that adds a xylosyl group to the hydroxyl group at C2’’. UGT84A2 is a sinapic acid: UDP-glucosyltransferase that catalyzes the formation of 1-*O*-sinapoylglucose by adding glucose to sinapic acid. The knockout mutation of *UGT84A2* lead to the reduction of the levels of A11, a dominant sinapoylated cyanin in wild-type (WT) Arabidopsis leaves [26]. This result suggests that 1-*O*-sinapoylglucose serve as a donor of sinapoyl moieties to form sinapoylated cyanins. The biochemical mechanism by which the glucose group is attached to the *p*-coumaroyl moiety on the anthocyanin structures remains to be elucidated. 

Acylation is another main biochemical mechanism leading to diverse anthocyanin molecules in Arabidopsis [25,28]. To date, several enzymes have been characterized to catalyze these acylation reactions. *At3g29590*, *At1g03940* and *At1g03495* have been identified to encode three BAHD types of anthocyanin acyltransferases (AATs) that use malonyl-CoA or *p*-coumaroyl-CoA as substrates to transfer the malonyl or *p*-coumaroyl groups to cyanin structures [24]. In addition, *At2g23000* has been characterized to encode a serine carboxypeptidase-like (SCPL) type of AAT. This enzyme has been shown to use sinapoylglucoses as substrates to transfer sinapoyl groups to cyanins to form sinapoylated cyanins [27]. 

Methylated forms of cyanin molecules have been detected from Arabidopsis [14,16,17]. Although, to date, genes encoding anthocyanin methyltransferases have not been characterized in Arabidopsis, several of them have been identified from other species such as petunia and grape [25]. *S*-adenosyl-L-methionine (SAM) dependent *O*-methyltransferases (OMTs) have been reported to be responsible for catalyzing the methylation of various natural products [25,29]. The methylation process of anthocyanins in Arabidopsis is unclear and whether there exist OMTs in Arabidopsis responsible for the formation of methylated anthocyanins remains to be elucidated. 

## BIOSYNTHETIC PATHWAY

The anthocyanin biosynthetic pathway is a major branch of the general phenylpropanoid pathway that starts with phenylalanine, Fig. (**2**). In general, from phenylalanine to anthocyanins, the biosynthetic pathway can be divided into three phases: beginning steps of the general phenylpropanoid pathway, early steps of the flavonoid pathway and late steps of the anthocyanin specific pathway. 

The beginning steps of the phenylpropanoid pathway include three consecutive steps from phenylalanine through cinnamic acid and coumaric acid to 4-coumaroyl CoA, which are catalyzed by phenylalanine ammonia-lyase (PAL), cinnamate-4-hydroxylase (C4H) and 4-coumaroyl CoA: ligase (4CL), respectively. In addition to flavonoid biosynthesis, these three steps of the phenylpropanoid pathway also lead to the production of hydroxycinnamic acid derivatives such as sinapate esters and monolignols. Genes encoding PAL, C4H and 4CL have been cloned and characterized from Arabidopsis. Four genes have been identified to encode isomers of PAL. Knockout mutant analyses and gene expression experiments under nitrogen depletion and low temperature conditions have shown that two isomers, *PAL1* and *PAL2*, are preferably involved in the flavonoid pathway [30-32]. A small gene family has been identified to encode 4CL in Arabidopsis. Studies of gene expression pattern and enzyme properties have revealed that 4CL3 appears to be preferably associated with the flavonoid pathway, while 4CL1 and 4CL2 are most likely involved in the formation of hydroxycinnamic acid derivatives [33]. In contrast to PAL and 4CL, only one gene in the Arabidopsis genome has been identified to encode C4H.

The early steps of the flavonoid pathway are from 4-coumaroyl CoA through chalcone and naringenin to dihydroflavonol. These three reaction steps are catalyzed by chalcone synthase (CHS), chalcone isomerase (CHI), flavanone 3-hydroxylase (F3H), respectively, and as a result, dihydrokaempferol characterized by a hydroxyl group at C4’ in the B-ring is produced. The subsequent hydroxylation of dihydrokaempferol at C3’ catalyzed by the flavonoid 3’-hydroxylase (F3’H) leads to the synthesis of dihydroquercetin. To date, dihydrokaempferol and dihydroquercetin are the only two dihydroflavonol molecules identified in Arabidopsis. Genes encoding these pathway enzymes have been biochemically and genetically characterized in Arabidopsis. Knockout mutations of these genes lead to the lack of production of both anthocyanins and proanthocyanidins in seeds resulting in transparent testa [34-36].

The late steps of the anthocyanin pathway include steps from dihydroflavonols through leucoanthocyanidins to anthocyanidins as well as the further modifications of anthocyanidins as described above. The steps from dihydroflavonols to anthocyanidins are consecutively catalyzed by dihydroflavonol reductase (DFR) and anthocyanidin synthase (ANS, also called leucoanthocyanidin dioxygenase, LDOX). These two enzymes are encoded by a single gene respectively. The knockout mutants of either of these two genes lead to transparent testa phenotypes in seeds [34,35]. In addition, as described above, modifications including glycosylation and acylation convert anthocyanidins to diverse anthocyanin molecules. 

## METABOLIC CHANNELING *IN VIVO*

Successive enzymes of the phenylpropanoid pathway are proposed to be grouped together and associated with the membrane of the endoplasmic reticulum (ER) to form protein complexes that direct the channeling of the intermediate precursors in the complex without diffusing to the cytosol [34,37,38]. Evidence for the channeling of intermediates and the co-localization of pathway enzymes has been reported [39]. In addition, direct *in vitro* studies have shown that PAL and C4H were co-localized on ER membranes of tobacco cells [40]. It has been hypothesized that the membrane-anchored C4H and F3’H, two members of the cytochrome P450 family proteins, might act as nucleation sites for the binding of other soluble enzymes to the complex [34,40]. Although evidence is limited, this hypothesis is considered as a favorable model for the synthesis and channeling of anthocyanins and other flavonoids.

## TRANSPORT AND COMPARTMENTATION

Anthocyanins are stored in the central vacuole of cells. As described above, the biosynthesis of anthocyanins takes place in the cytosol. Anthocyanins need to be transported from the cytosol to the vacuole. Transporter-mediated and vesicle-mediated transport are two major hypotheses proposed for the transport of anthocyanins to the vacuole [23,41,42]. 

In general, the hypothesis of transporter-mediated transport is supported by the identification of flavonoid transporters involved in the vacuolar transport of specific types of anthocyanins and proanthocyanidin precursors in different plant species [43-46]. In Arabidopsis, three genes, *TT12*, *TT19* and *AHA10*, have been functionally characterized to be associated with the transport of anthocyanins. *TT12* encodes a multidrug and toxic efflux (MATE) antiporter that has been demonstrated to be responsible for the vacuolar uptake of glycosylated flavan-3-ols and possibly glycosylated anthocyanidins in the endothelial cells of seeds [43,47]. The *tt12* mutants lack the formation of proanthocyanidins in seeds and show a transparent testa phenotype. Also, the endothelial cells of *tt12* mutants form multiple vesicles instead of a large central vacuole. *AHA10* encodes a plasma membrane H^+^-ATPase that has been reported to likely function in endosomal or vacuolar compartments [48]. The *aha10* knockout mutants are characterized by transparent testa of seeds as well. Endothelial cells in seed coat of this mutant do not develop the central vacuole; instead, produce numerous vesicles filled with epicatechin molecules that are precursors of proanthocyanidins. Experiments have shown that AHA10 is essential for the acidification of the central vacuole and the formation of the proton gradient necessary for the function of TT12 in the seed endothelial cells. Given that *TT12* and *AHA10* are primarily expressed in developing seeds, these two genes likely co-ordinate the subcellular transport and compartmentation of anthocyanins and proanthocyanidins in the seed coat. The mechanism of the vacuolar uptake of anthocyanins in vegetative tissues remains unclear. It has been hypothesized that homologs of *TT12 *likely function in vegetative tissues to mediate the transport of anthocyanins [43]. In addition, homologs of the multidrug resistance-associated protein (MRP) type of ABC transporters similar to the ZmMRP3 in maize [44] are also potential candidates involved in anthocyanin transport from the cytosol to the large central vacuoles in vegetative tissues. *TT19* encodes a glutathione *S*-transferase (GST) that has been demonstrated to be involved in the vacuolar uptake of both anthocyanins and proanthocyanidin precursors [49]. The *tt19 *mutants lack the production of proanthocyanidins in the seed coat and show transparent testa phenotypes. TT19 was proposed to function as a carrier protein to ‘escort’ anthocyanins or proanthocyanidin precursors to the vacuole [34,42,49-51]. *In vitro* biochemical analysis has shown that TT19 has a very weak GST activity, and no anthocyanin-glutathione conjugates have been detected in Arabidopsis [34,49,51,52]. A recent study demonstrated that TT19 can bind to not only cyanidin but also to cyanidin 3-*O*-glucoside, although the affinity to the latter is lower than to the former [53]. Based on the cytosolic localization of TT19, the binding of TT19 to cyanidin most likely occurs near the cytosolic surface of ER. TT19 might function in protecting cyanidin from degradation during the transport process. Furthermore, given that recently the TT19 fusion protein was observed to be localized in the tonoplast as well [53], it likely has additional functions that needs further characterization. 

The evidence for vesicle-mediated transport results from the observation of cytoplasmic vesicle-like structures filled with anthocyanins and the anthocyanic vacuolar inclusions (AVIs) that exist in the large central vacuole [23,41]. This mechanism can be indirectly supported by the formation of small vesicles instead of a large central vacuole in the seeds of *aha10* and *tt12* mutants as described above. These phenotypes also suggest that the transporter-mediated and vesicle-mediated mechanisms may act in concert to direct the transport of anthocyanins. 

## METABOLIC ENGINEERING OF ANTHOCYANINS *IN VITRO*

The isolation of anthocyanin-producing cells *in vitro* from Arabidopsis has not been reported until recently. We established anthocyanin-producing cell lines through tissue culture from rosette leaves of *pap1-D* plants [17,54]. On a modified MS medium (without NH_4_NO_3_ and with half-strength KNO_3_) supplemented with 0.1 mg L^-1^ 2,4-dichlorophenoxyacetic acid (2,4-D) and 0.25 mg L^-1^ kinetin, red calli were selected and maintained. During *in vitro* selection, metabolic differentiation occurred in cultured cells. As a result, several red cell lines with different anthocyanin levels were developed. In addition, anthocyanin-free cells from *pap1-D* plants were also established. Microarray and RT-PCR analysis showed up-regulation of the expression of most late pathway genes as well as transcription factors including *PAP1*, *TT8* and *GL3* in red *pap1-D* cells. LC-MS based profiling identified seven cyanin molecules from red *pap1-D* cells. The anthocyanin-producing *pap1-D* cells provide an appropriate model system to understand the mechanisms of how other factors control the activities of the WBM complexes discussed below. 

## TRANSCRIPTIONAL REGULATION OF PATHWAY GENES

Over the past two decades, the regulation of the anthocyanin biosynthetic pathway has gained intensive investigations in Arabidopsis [55,56]. Pathway genes of flavonoid biosynthesis were shown to be co-regulated [15,18,55,57]. Particularly, studies of mutants, gene expression profiling, protein-DNA and protein-protein interactions have shown that the expression of late biosynthetic genes of anthocyanins is regulated by a ternary WD40-bHLH-MYB (WBM) complex composed of MYB, bHLH and WD40 transcription factors.

Four MYB transcription factors, PAP1/ MYB75, PAP2/ MYB90, MYB113 and MYB114 with relatively high sequence similarities, have been identified to control anthocyanin biosynthesis in vegetative tissues. All these four genes are R2R3-MYB proteins that contain two imperfect repeats in the MYB domain [58,59]. *PAP1* (*Production of Anthocyanin Pigmentation 1*) was identified by T-DNA activation tagging [19]. The overexpression of *PAP1* in *pap1-D *activation tagging lines and *35S:PAP1* transgenic plants leads to high accumulation of anthocyanins in leaves, stems, flowers and roots [15,18,19]. In addition, the overexpression of *PAP2*, *MYB113* and *MYB114 *also leads to an increase in anthocyanin production [19,60]. In contrast, the *pap1* knockout mutants and the knockdown plants of *PAP1*, *PAP2*, *MYB113* and *MYB114* by RNAi lack anthocyanins in leaves and seedlings [60]. Gene expression analysis has shown that the expression of *DFR* and *ANS *is highly activated in plants overexpressing these genes [15,16,18,19,60], but reduced or inactivated in *pap1* knockout mutants and *PAP1* RNAi knockdown plants [60]. Among the four MYB transcription factors, it appears that PAP1 is a master regulator of anthocyanin biosynthesis. *PAP1 *is expressed at the highest level in comparison with its homologs. The metabolic engineering of red *pap1-D* cells has demonstrated that the overexpression of *PAP1* alone can activate the anthocyanin pathway especially the expression of late pathway genes [17,54,61]. In addition, the overexpression of *PAP1* in several other plant species has resulted in obvious increases in anthocyanin levels [62-66]. These data show that PAP1 is a key regulator controlling the biosynthesis of anthocyanins. It is hypothesized that PAP2, MYB113 and MYB114 might be specialized in regulating anthocyanin biosynthesis under certain conditions or at specific developmental stages of plants.

Three members of the bHLH transcription factor family, GL3 (Glabra 3), EGL3 (Enhancer of Glabra 3) and TT8 (Transparent testa 8), have been identified to positively regulate anthocyanin biosynthesis. Based on the classification of the bHLH protein family, these three members belong to the subgroup IIIf [67-70]. These three homologs are not simply functionally redundant. In contrast, they have overlapping but distinct functions in regulating several physiological and developmental processes, such as trichome initiation, non-root hair cell fate determination, seed coat mucilage formation, anthocyanin and proanthocyanidin biosynthesis [60,71-75]. *GL3* and *EGL3* were identified from the phenotypes of their knockout mutants. In Arabidopsis, gene expression and biochemical analysis have shown that GL3 and EGL3 were essentially associated with trichome development, pavement cell fate determination and cell patterning. In particular, promoter activity analyses have shown that the expression of these two genes spatially occurs in mature embryos, expanding cotyledons, root tips, leaf primordium and young seedlings [55,60,74,75]. The function of GL3 in regulating anthocyanin biosynthesis was first observed in a transient expression experiment, in which the co-expression of *GL3* and *MYC-146* led to the formation of anthocyanins in white flower mutants of *Matthiola incana* [76]. The involvement of GL3 in anthocyanin biosynthesis subsequently was supported by mutant analysis and gene expression studies. The pigmentation of anthocyanins in the cotyledon and hypocotyl of seedlings was phenotypically lower in *egl3*, *gl3* and *egl3* *gl3* mutants than in wild-type plants. The *egl3* *gl3* mutants lost the most reddish pigmentation, followed by *egl3* and then *gl3* mutants [75]. In addition, the overexpression of *EGL3 *in the *ttg1* mutant background resulted in more anthocyanin pigmentation than the overexpression of *GL3 *in the same mutant background [75]. These two observations were supported by results from inducible gene expression experiments. In brief, the expression of the recombinant *GL3* induced by dexamethasone in *gl3* and *gl3 egl3* mutant backgrounds revealed that when EGL3 was present, the gene expression levels of *DFR* and *ANS *were similar no matter whether GL3 was present or not [60]. These observations suggested that EGL3 had a stronger regulatory activity on anthocyanin biosynthesis than GL3 in seedlings [60]. However, the regulatory function of GL3 in anthocyanin biosynthesis was also shown by experiments testing the effects of nitrogen depletion. This study revealed the involvement of *GL3* but not *EGL3* in the formation of anthocyanins in rosette leaves under nitrogen deficient conditions [77]. The result seems to be controversial to the previous observations about the relative contribution of GL3 and EGL3 on anthocyanin biosynthesis, but this difference might be explained by different experimental materials and/or treatments used in the studies. Taken together, all these experiments indicated that the involvement of EGL3 in the regulation of anthocyanin biosynthesis is likely conditional; GL3 and EGL3 might have functional specificity under different developmental stages and/or environmental conditions. From mutant analysis, the locus *TT8* was first identified to encode a transcription factor [35]. The seeds of this mutant lack the brownish pigmentation produced by oxidation of proanthocyanidins, but anthocyanin biosynthesis was only moderately affected in young seedlings and leaves. The subsequent gene cloning and characterization demonstrated that *TT8* encoded a bHLH protein regulating the expression of *DFR*, *ANS* and *BAN* (*ANR*) in the endothelial layer of seed coat [73]. Its expression was detected in seedlings, buds, flowers, and developing siliques, but barely detectable in rosette leaves, stems and roots [73]. Promoter analysis also revealed the expression pattern of *TT8* in developing siliques and young seedlings [78] as well as in the main veins of rosette leaves [79]. We recently isolated red cells from tissue culture of *pap1-D *rosette leaves overexpressing *PAP1*. Comparative qRT-PCR and microarray analyses showed a strong up-regulation of *TT8* in red *pap1-D* cells [17]. All data have suggested that TT8 not only regulates anthocyanidin production towards the synthesis of proanthocyanidins in seeds, but is also involved in the regulation of anthocyanin biosynthesis in vegetative tissues and cell cultures. In addition, EGL3 and TT8 have been identified to have a shared role in regulating seed coat mucilage production [75]. Moreover, studies have shown that *TT8* expression can be controlled by several MYB and bHLH transcription factors. The expression of *TT8* is increased in transgenic plants overexpressing *PAP1* or *TT2 *[78]. In the *gl3 egl3* mutant background, *TT8* promoter has been shown to have a lower activity than in wild-type plants, indicating the necessity of GL3 and EGL3 in controlling the expression of *TT8 *[78]. Also, TT8 has been shown to be able to regulate its own expression [78]. Although most of the investigations have not reported the involvement of TT8 in epidermal cell fate determination during normal growth of plants, a recent report showed that TT8 was involved in the development of marginal trichomes of rosette leaves treated with jasmonic acid (JA), 6-benzylaminopurine (BAP) and gibberellic acid (GA) [80].

TTG1 is the only WD40 protein member currently determined to regulate anthocyanin biosynthesis in Arabidopsis. Mutation in the *TTG1* locus results in pleiotropic impacts on plant development and metabolism, including the deficiency of anthocyanin production in vegetative tissues, the deficiency of proanthocyanidins in seed coat and defects in trichome initiation, non-root hair cell fate determination and seed mucilage production [35,81,82]. Multiple experiments have demonstrated that TTG1 is constitutively expressed in all tissues throughout the entire development of plants; in addition, its expression does not respond to alteration of environmental conditions tested [83-85]. All current data have shown that TTG1 has a central role in the WBM regulatory complexes to regulate epidermal cell fate and metabolic specificity leading to the production of anthocyanins and proanthocyanidins. 

## THE WD40/BHLH/MYB REGULATORY COMPLEXES

It has been shown that the activation of anthocyanin biosynthetic pathway, especially late biosynthetic steps in Arabidopsis, is controlled by a ternary complex formed by WD40, bHLH and MYB transcription factors, including TTG1, GL3, EGL3, TT8, PAP1, PAP2, MYB113 and MYB114 described above. The WD40/bHLH/MYB (WBM) complexes controlling anthocyanin biosynthesis have been identified from other plant species as well such as maize and petunia [55,56]. To date, TTG1 has been demonstrated to play a central role in the regulatory network in all WBM complexes potentially identified. The function of TTG1 in the WBM complex has been suggested to stabilize the protein-protein interactions [56,86]. The WD motifs in TTG1 are normally the sites responsible for interacting with other proteins. TTG1 has been found to be required for the normal distribution of GL3 in the nucleus. The loss of TTG1 caused the GL3-YFP protein to be distributed abnormally in the nucleus resulting in ‘speckles’ [74]. Also, a recent study demonstrated that nuclear-localized GL3 can recruit TTG1 to the nucleus by interacting with the TTG1 protein [87]. 

Protein-protein interactions among bHLHs, MYBs and TTG1 have been demonstrated by different experiments. Yeast two-hybrid and pull down assays have provided evidence that GL3, EGL3 and TT8 interact with TTG1, MYB family proteins PAP1/PAP2 and bHLH proteins themselves [71,75,88]. In addition, TT8 has been demonstrated to interact with TT2 and TTG1 to regulate proanthocyanidin biosynthesis [71]. GL3 and EGL3 also interact with GL1 and WER, which are involved in the regulation of trichome initiation and non-root hair cell fate determination, respectively [75,88]. Sequence analysis revealed a conserved motif consisting of [DE]Lx_2_[RK]x_3_Lx_6_Lx_3_R in the R3 repeat of MYB proteins interacting with bHLHs. Site mutation studies confirmed that this motif is responsible for the interaction with bHLH proteins [88]. Those R2R3-MYBs such as MYB11, MYB12 and MYB111 that do not contain this motif have been demonstrated to be unable to interact with bHLHs. Two transient expression experiments have also indicated that MYB proteins interact with bHLH proteins to activate the transcription of late biosynthetic genes [88,89]. 

The C-terminal region of bHLH proteins is suggested to be required for regulating anthocyanin biosynthesis. Sequence analysis has identified that the N-terminal of bHLH proteins contains a region interacting with MYB and an acidic region while the C-terminal includes a bHLH domain likely involved in the formation of homodimer and heterodimer [56,90]. A recent study showed that the ectopic expression of the N-terminal region of the maize R protein, a homolog of GL3 and EGL3, can regulate leaf trichome and root hair differentiation in Arabidopsis, but for activating anthocyanin biosynthesis, the full length R is required [91]. These data suggest that the dimerization of the bHLH proteins is likely a prerequisite for activating anthocyanin biosynthesis. It is also possible that other co-factors might be recruited to the C-terminal regions of bHLH proteins and are required for the activation of target genes. In maize, an EMSY-related factor involved in the histone modification has been found to specifically interact with the bHLH region of R and is necessary for the activation of the expression of anthocyanin biosynthetic genes [92].

The component properties of different WBM complexes remain to be further elucidated. Although biochemical and genetic studies have shown that TTG1 (WD40), GL3/ EGL3/TT8 (bHLH) and PAP1/PAP2/MYB113/MYB114 (MYB) are components of potential WBM complexes [54,60]. The understanding of how many bHLH and MYB members are necessary to form a functional complex to activate the biosynthesis of anthocyanins at different developmental stages and environmental conditions is limited. To date, it appears that only the WBM complex in endothelial layers of seed coats has been determined to consist of TTG1, TT8 and TT2 [71]. In comparison, the components of bHLHs and MYBs in leaves are more complicated. Gene expression studies and protein profiles in single cells or in metabolically and morphologically identical cells in leaves might be helpful. We recently isolated red cells from rosette leaves of *pap1-D* plants and cultured them *in vitro*. Although these red cells were characterized by heterogeneity of pigmentation, no specific morphological differentiations, such as the formation of pavement cells and trichomes, occurred in the culture conditions [17]. Genome-wide gene expression analysis identified the up-regulation of *PAP1* and *TT8* in red cells. In addition, qRT-PCR analysis showed the up-regulation of the expression of *GL3* in red cells. Our experiments suggest that TTG1, GL3/TT8 and PAP1 likely form the only WBM complex that activates the high production of anthocyanins in engineered *pap1-D* cells. 

In Arabidopsis, in addition to regulating anthocyanin biosynthesis, WBM complexes are involved in the control of other physiological and developmental processes including trichome initiation, non-root hair cell fate determination and mucilage production in the seed coat. This is in contrast to the function of WBM complexes in maize which seems to only control anthocyanin biosynthesis [55,56,71,74]. The involvement of WBM complexes in such broad aspects of cellular events in Arabidopsis likely results from the overlapping but distinct functions of multiple members of bHLH and MYB proteins as discussed above.

## REGULATION OF THE ACTIVITIES OF WBM COMPLEXES BY FACTORS *IN PLANTA*

The activities of WBM complexes can be affected by factors *in planta*. As described above, TTG1, bHLHs (GL3/ EGL3/TT8) and MYBs (PAP1/PAP2/MYB113/MYB114) can form different WBM complexes to activate anthocyanin biosynthesis. However, other proteins, such as CPC and MYBL2 which are 1R-MYB members, have been demonstrated to negatively control the activities of WBM complexes resulting in the decrease in the biosynthesis of anthocyanins shown in Fig. (**3**). Results from transient expression and protein interaction studies have suggested that CPC and MYBL2 compete with positive regulators PAP1/PAP2 to bind bHLH proteins and interfere with the formation of active WBM complexes thus negatively regulating the expression of pathway genes [79,88,89]. In addition to anthocyanin biosynthesis, CPC was identified to negatively regulate trichome initiation and non-root hair cell fate determination [93,94]. The overexpression of *MYBL2* has been shown to repress trichome development [95]. The C-terminal of MYBL2 contains a repression domain composed of TLLLFR that has been shown to have a strong repressive activity [79]. A recent study has shed some light on the mechanisms in determining epidermal cell fate. The results revealed that the cell fate of root epidermal cells is determined by the quantitative competition between the levels of the positive R2R3-MYB regulator WER and the negative 1R-MYB protein CPC [96]. We propose that a similar regulatory mechanism might also control the production of anthocyanins in Arabidopsis cells. The quantitative competition between positive regulators PAP1/PAP2/MYB113/ MYB114 and negative regulators CPC/MYBL2 may determine the activation/repression of the expression of pathway genes, Fig. (**3**).

Small regulatory RNAs were recently uncovered to control anthocyanin biosynthesis through a mechanism of regulating the expression of the members of the WBM complexes. *TAS4*-siR81(-), which is derived from *TAS4* and *miR828, *is a trans-acting siRNA. *TAS4*-siR81(−) and *miR828* were shown to target PAP1/PAP2/MYB113 [97,98]. In phosphate deficient conditions, the expression level of *PAP1* is increased in tissues. PAP1 has been demonstrated to activate the expression of *TAS4 *and *miR828*, which may further function in a feedback manner to target *PAP1* and its homologs to reduce their expression [97]. These results have revealed a potential autoregulatory mechanism of *PAP1* expression through *TAS4*-siR81(−) and *miR828*. In addition, transgene silencing of *PAP2* has been observed in homozygous transgenic tobacco plants and was suggested to be caused by small regulatory RNAs similar to *TAS4*-siR81(−) and *miR828 *in Arabidopsis [99]. Another example of small RNA involved in the regulation of anthocyanin biosynthesis is *miR156*. The *SQUAMOSA PROMOTER BINDING PROTEIN*-*LIKE* (*SPL*) transcription factor targeted by *miR156* has been demonstrated to negatively regulate the acropetal accumulation of anthocyanins in the inflorescent stem [100]. SPL9 was observed to be able to interact with PAP1 and can directly bind to the promoter of *DFR*. SPL9 was suggested to negatively control the expression of anthocyanin pathway genes by competing with bHLH proteins for binding with PAP1. The high expression of *miR156* indirectly positively regulates the expression of anthocyanin pathway genes. 

## REGULATION OF ANTHOCYANIN BIOSYNTHESIS BY ABIOTIC FACTORS AND PHYTOHORMONES

Anthocyanin biosynthesis can be induced by various abiotic factors such as high light, low temperature, sucrose, nutrient depletion and phytohormones [15,77,84,101-105]. Numerous significant advances have been made in elucidating the molecular mechanisms of anthocyanin biosynthesis in response to these factors, several of which are summarized below. 

### Light 

Light is one of the most important environmental factors affecting biosynthesis of anthocyanins. Strong light conditions can increase the production of anthocyanins [15,16,83]. In contrast, dark conditions can lead to the decrease of anthocyanins. Although the mechanism of light regulation on anthocyanin biosynthesis remains to be completely elucidated, many studies have demonstrated that the expression of pathway and regulatory genes involved in anthocyanin biosynthesis is controlled by different light conditions. As multiple experiments have shown, all pathway genes are expressed in seedlings and rosette leaves of Arabidopsis plants in strong light conditions [15,16,83]. Also, it has been shown that the activation of these pathway genes in light conditions is likely through controlling the expression of the members of the WBM complexes [15,16,83]. For example, the expression of *PAP1*, *PAP2* and *bHLH* genes *GL3*, *EGL3* and *TT8* were all induced by various light spectra [83]. As described above, PAP1 is a master regulator of anthocyanin biosynthesis. Nevertheless, several studies showed that *PAP1* overexpression alone was not sufficient for the activation of anthocyanin biosynthesis in the dark or under low light conditions [15,16,83], which suggests that the accumulation of other factors such as bHLH or HY5 proteins in response to light is needed to activate anthocyanin pathway gene expression. 

In addition, light signaling components have been demonstrated to play important roles in controlling anthocyanin biosynthesis. HY5, a bZIP protein, is a positive regulator of photomorphogenesis and can be degraded by COP1 in dark-grown seedlings [106]. In far-red light conditions, HY5 and PIF3 (a phytochrome interacting factor) collaboratively regulate anthocyanin biosynthesis in germinating seedlings. HY5 and PIF3 can simultaneously bind to different sequence elements in the promoters of several anthocyanin pathway genes and positively regulate their expression [107]. In addition, HY5 has been demonstrated to be a key effector in the UV light signaling pathway that was mediated by UV RESISTANCE LOCUS8 (UVR8) [108] and also in the cryptochrome photoreceptor-mediated blue light response [109]. The light-regulated zinc finger protein 1 (LZP1), which functions in the downstream of HY5, has also been identified to act as a positive regulator in de-etiolation. LZP1 has been shown to positively regulate anthocyanin biosynthesis through a mechanism of directly or indirectly controlling the expression of *PAP1* [110]. Furthermore, light regulatory units (LRUs) sufficient for light responsiveness have been identified in the promoters of the *CHS*, *CHI*, *F3H* and *FLS* genes in studies conducted under UV-containing white light. The LRUs have been characterized to include a MYB-recognition element (MRE) and an ACGT-containing element (ACE), the latter of which is recognized by bZIP proteins such as HY5 [111]. 

### Sucrose

Sucrose has been demonstrated to regulate anthocyanin biosynthesis in plants and cell cultures. In general, treating Arabidopsis seedlings with increased levels of sucrose can enhance the production of anthocyanins [104]. A time course study of gene expression has shown that most pathway genes are induced in seedlings treated with sucrose [103]. The increase of pathway gene expression most likely results from the induction of *PAP1*. A QTL analysis has shown that the expression of *PAP1* is responsible for sucrose-induced anthocyanin accumulation [104]. In addition, a microarray study on seedlings treated with sucrose versus controls has revealed a strong up-regulation of *PAP1* but not *PAP2* [103].

Sucrose transporters appear to play a role in sucrose-induced anthocyanin biosynthesis. The mutants of *SUC1* (*SUCROSE TRANSPOTER1*) showed less anthocyanin accumulation in response to sucrose [112]. In addition, *SUC2*, a homolog of *SUC1*, has been shown to be involved in anthocyanin production in conditions of phosphate deficiency. The expression of *SUC2* is highly up-regulated in the *hypersensitive to phosphate starvation1* (*hps1*) mutant, which has an enhanced sensitivity to phosphate starvation [113]. Consequently, in this mutant, the levels of sucrose are much higher than in wild-type plants. As a result, the seedlings of *hps1* mutants have enhanced production of anthocyanins. 

In addition, a crosstalk between sucrose and plant growth regulators has been shown to regulate anthocyanin biosynthesis. Jasmonate and cytokinin are known to induce anthocyanin production in plants; however, in the absence of sucrose, the regulatory functions of these plant hormones are not obvious [105,114]. Ethylene has been observed to suppress the sucrose-induced anthocyanin biosynthesis. One mechanism is that ethylene treatments lead to the down-regulation of the expression of *GL3*, *TT8* and *PAP1 *[115,116]. In addition, ethylene treatments cause the down-regulation of *SUC1* in roots [115]. 

### Nitrogen

Nitrogen sources can strongly control the biosynthesis of anthocyanins in Arabidopsis. A general trend is that seedlings produce low levels of anthocyanins in high concentrations of total nitrogen, in contrast, high levels of anthocyanins in low concentrations of nitrogen. Under nitrogen deficient conditions, seedlings have been reported to accumulate high levels of both anthocyanins and flavonols [84,85]. Pathway genes and regulatory genes have been shown to be regulated in response to nitrogen treatment. Transcriptional analyses have revealed that nitrogen depletion conditions induced the expression levels of *PAP1* and *PAP2 *[84,117]. In comparison, *PAP2* was shown to have a stronger response to nitrogen limitation than *PAP1*. This observation was supported by another experiment, in which the expression of *PAP2* was strongly induced in senescing leaves treated by high sugar/nitrogen ratios [118]. For three *bHLH* genes, *GL3* but not *EGL3 *was highly up-regulated in rosette leaves of wild-type plants under nitrogen depletion [84]. In contrast, the *gl3* mutants accumulate much lower amounts of anthocyanins in rosette leaves under nitrogen depletion conditions compared with WT and *egl3* mutants. A recent study suggested that the *FRUITFULL (FUL) *gene is also likely involved in the regulation of anthocyanin biosynthesis in response to nitrogen. The *FUL *gene regulates cell differentiation during fruit and leaf development in Arabidopsis [119]. Its homolog *VmTDR4* has been identified to be an important regulatory gene in regulating anthocyanin accumulation during the ripening of bilberry fruits [120]. Gene expression analysis revealed that FUL is necessary for the expression of *PAP2* under nitrogen depletion conditions [120]. Moreover, three LATERAL ORGAN BOUNDARY DOMAIN (LBD) family proteins, LBD37, LBD38 and LBD39, were recently identified to negatively regulate anthocyanin biosynthesis under nitrogen sufficient conditions [121]. The overexpression of these genes strongly suppressed anthocyanin production in plants grown under a nitrogen depletion condition. In contrast, the knockout mutants of these three genes accumulated high levels of anthocyanins even though grown under a nitrogen sufficient condition. Transcriptional analysis has revealed that these three regulators repress anthocyanin biosynthesis through suppressing the expression of *PAP1 *and *PAP2* [121]. 

### Jasmonate

Jasmonate (JA) is an elicitor and signal molecule that mediates plant responses to pathogen infection, UV radiation and other abiotic stresses [122]. JA can strongly increase anthocyanin biosynthesis in Arabidopsis. A recent study showed that the F-box protein COI1 was required for the expression of late anthocyanin biosynthetic genes as well as the regulatory genes *PAP1*, *PAP2* and *GL3* in response to JA [105]. It has been demonstrated that the COI1 protein interacts with ASK1/ASK2, Cullin1, and Rbx1 to form the SCF^COI1^ complex, which mediates the degradation of JA ZIM-domain (JAZ) proteins [123]. JAZ proteins have been shown to repress diverse JA responses including anthocyanin biosynthesis [124]. The potential mechanism is that JAZ proteins can interact with the C-terminal regions of both bHLH (TT8, GL3 and EGL3) and MYB (PAP1 and GL1) transcription factors to interfere the formation of active WBM complexes [125]. These results provide an appealing model for the molecular mechanism of JA-induced anthocyanin production, in which JA induces the degradation of JAZ proteins through the SCF^COI1^ complex, thus allowing the formation of the functional WBM complexes and leading to the production of anthocyanins. 

## Figures and Tables

**Fig. (1):**
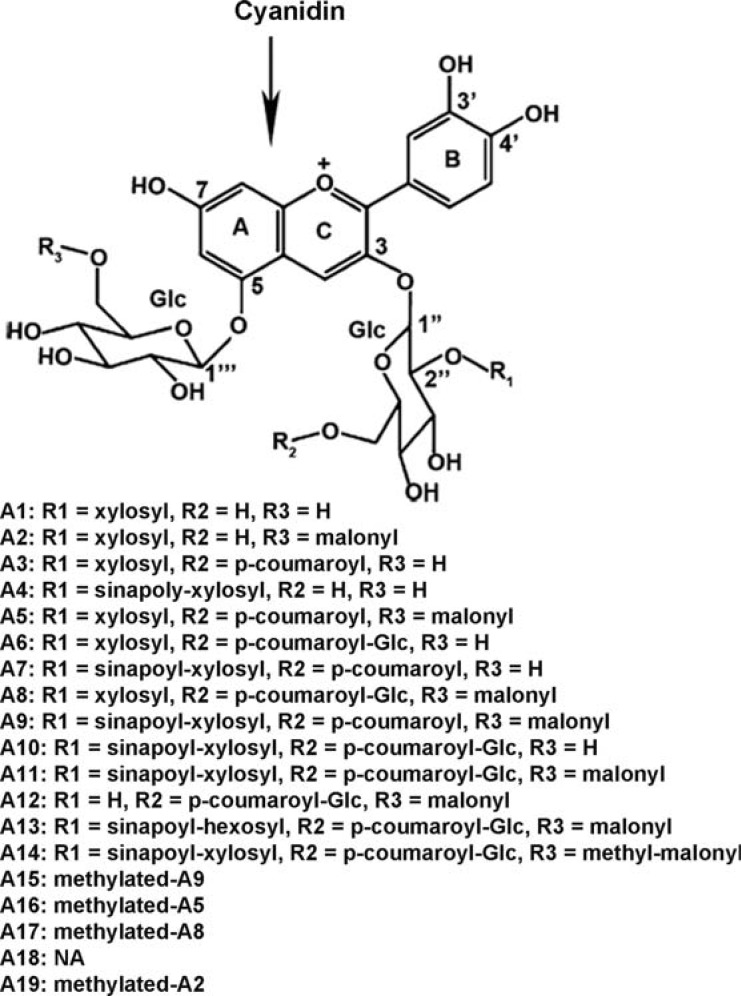
Structures of major anthocyanin molecules identified from Arabidopsis. Scheme modified from ref. [16]. A14-A19 molecules are deduced structures based on MS analysis. NA: not available due to the lack of report on MS fragments.

**Fig. (2):**
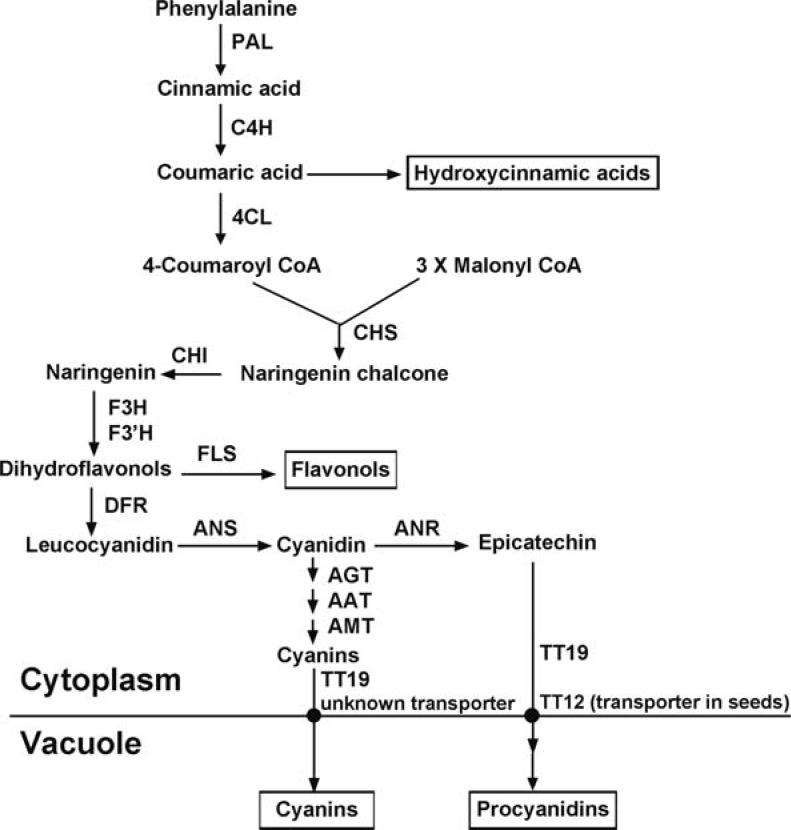
Anthocyanin biosynthetic pathway in Arabidopsis. Enzymes catalyzing corresponding steps are indicated. Related branches of the pathway leading to the production of other phenylpropanoid compounds are also indicated. PAL, phenylalanine ammonia lyase; C4H, cinnamate 4-hydroxylase; 4CL, 4-coumaroyl: CoA-ligase; CHS, chalcone synthase; CHI, chalcone isomerase; F3H, flavanone 3-hydroxylase; F3’H, flavonoid 3’-hydroxylase; DFR, dihydroflavonol reductase; ANS, anthocyanidin synthase; FLS, flavonol synthase; ANR, anthocyanidin reductase; AGT, anthocyanin glycosyltransferase; AAT, anthocyanin acyltransferase; AMT, anthocyanin methyltransferase; TT19, Transparent Testa 19; TT12, Transparent Testa 12.

**Fig. (3):**
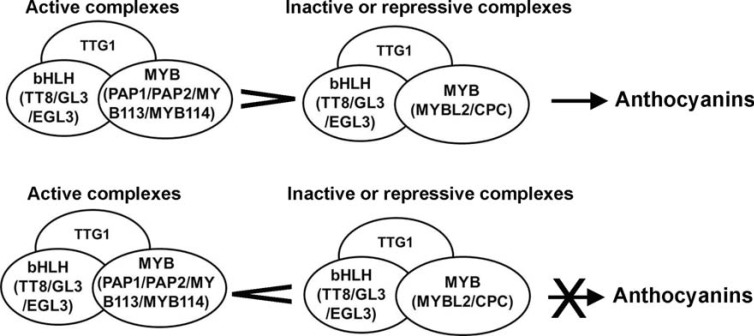
Regulation of anthocyanin production in Arabidopsis cells by quantitative competition between active WBM complexes and inactive or repressive WBM complexes.

**Table 1.** Major anthocyanin molecules identified from *Arabidopsis thaliana*.

Anthocyanin	ESI-MS	Reference about NMR Data	Detected Distribution in Tissues
A1	743	NA	Leaves and roots
A2	829	NA	Leaves, roots and callus cultures
A3 a	889	[20] b	Leaves, roots and callus cultures
A4	949	NA	Leaves
A5 a	975	[20] b	Leaves, roots and callus cultures
A6 a	1051	[20] b	Leaves
A7 a	1095	NA	Leaves
A8 a	1137	[21]	Leaves and roots
A9 a	1181	[22] c	Leaves
A10 a	1257	[21]	Leaves
A11 a	1343	[14]	Leaves
A12 a	1005	NA	Leaves
A13 a	1373	NA	Leaves
A14	1357	[14]	Leaves
A15	1195	NA	Leaves
A16	989	NA	Leaves and callus cultures
A17	1151	NA	Leaves
A18	1035	NA	Leaves
A19	843	NA	Callus cultures

^a^ both trans and cis isomers were detected. ^b^NMR data of the same molecule identified in the garden plants of Cruciferae. ^c^NMR data of the same molecule identified in *Matthiola Incana*. NA: not available.

**Table 2.** List of anthocyanin modification genes identified in *Arabidopsis thaliana*.

AGI No.	Gene Name	Annotation	Reference
Glycosyltransferase			
At5g17050	*UGT78D2*	Flavonoid 3-*O*-glucosyltransferase	[18]
At4g14090	*UGT75C1*	Anthocyanin 5-*O*-glucosyltransferase	
At5g54060	*UGT79B1*	Anthocyanin 3-*O*-glucoside: 2’’-*O*-xylosyltransferase	[26]
At3g21560	*UGT84A2*	Sinapic acid: UDP-glucosyltransferase	
Acyltransferase			
At3g29590	*A5G6’’’MaT*	Anthocyanin 5-*O*-glucoside:6’’’-*O*-malonyltransferase	[24]
At1g03940	*A3G6’’p-CouT*	Anthocyanin 3-*O*-glucoside:6’’-*O*-*p*-coumaroyltransferase	
At1g03495	*A3G6’’p-CouT*	Anthocyanin 3-*O*-glucoside:6’’-*O*-*p*-coumaroyltransferase	
At2g23000	*SCPL10*	Sinapoylglucose:anthocyanin acyltransferase	[27]
Methyltransferase (unknown)			

**Table 3.** Major relevant patents regarding the regulation and manipulation of anthocyanin production in plants.

Patent #	Title	Year of Patent
US 6573432-B1	Regulation of anthocyanin pigment production [126]	2003
US 7973216-B2	Compositions and methods for modulating pigment production in plants [127]	2011
US 20100319091-A1	Methods of modulating production of phenylpropanoid compounds in plants [128]	2010
US 20090100545-A1	Means and methods to modulate flavonoid biosynthesis in plants and plant cells [129]	2009
US 8008543-B2	Modification of flavonoid biosynthesis in plants by PAP1 [130]	2011
US 7960608-B2	Modification of flavonoid biosynthesis in plants [131]	2011
US 20100186114-A1	Modification of plant flavonoid metabolism [132]	2010

## CURRENT AND FUTURE DEVELOPMENTS

To date, numerous accomplishments of studies in understanding anthocyanin biosynthesis and in metabolic engineering have led to the development of several patents. Major relevant patents regarding the regulation and manipulation of anthocyanin production in plants are listed in (Table **3**). These technologies will likely enhance the further engineering of novel anthocyanin molecules with high nutritional values in plants to benefit human health.

In spite of the numerous progresses made in elucidating the biosynthetic and regulatory process of anthocyanin biosynthesis, many questions still remain unanswered in this research area. For example, how do WBM complexes respond to different environmental factors? Are there functional specificities for different WBM complexes? How do negative transcription factors interact with WBM complexes? What occurs in the crosstalk between different regulatory complexes? Also, enzymes involved in the synthesis of different anthocyanin molecules remain to be comprehensively elucidated; regulatory mechanisms of tissue specific profiles of anthocyanin molecules need to be further investigated. Most of the current studies have been completed on young seedlings after seed germination. To comprehensively understand anthocyanin biosynthesis, plants grown at different growth stages and under various environmental conditions need to be investigated. To accurately understand the regulatory mechanisms, single cells or metabolically and morphologically identical cells such as anthocyanin-producing *pap1-D* cells as reported by Shi and Xie (2011) are helpful systems to elucidate the mechanism of environmental regulation of anthocyanin biosynthesis and the functional specificity of WBM complexes.

## ABBREVIATIONS

4CL: = 4-coumaroyl: CoA-ligase
ANS: = anthocyanidin synthase
bHLH: = basic helix-loop-helix
C4H: = cinnamate 4-hydroxylase
CHI: = chalcone isomerase
CHS: = chalcone synthase
COI1: = coronatine insensitive 1
COP1: = constitutive photomorphogenic 1
CPC: = caprice
DFR: = dihydroflavonol reductase
EGL3: = enhancer of glabra 3
ESI-MS: = electrospray ionization mass spectrometry
F3H: = flavanone 3-hydroxylase
F3'H: = flavonoid 3'-hydroxylase
GL3: = glabra 3
MYBL2: = MYB-like 2
NMR: = nuclear magnetic resonance
PAL: = phenylalanine ammonia lyase
PAP1: = production of anthocyanin pigment 1
TTG1: = transparent testa glabra 1
WBM: = WD40-bHLH-MYB
TT8: = transparent testa 8
